# Ventilation-Like Mechanical Strain Modulates the Inflammatory Response of BEAS2B Epithelial Cells

**DOI:** 10.1155/2019/2769761

**Published:** 2019-06-19

**Authors:** Sashko G. Spassov, Christoph Kessler, Rebecca Jost, Stefan Schumann

**Affiliations:** Department of Anesthesiology and Critical Care, Medical Center-University of Freiburg, Faculty of Medicine, University of Freiburg, Germany

## Abstract

Protective mechanical ventilation is aimed at preventing ventilator-induced lung injury while ensuring sufficient gas exchange. A new approach focuses on the temporal profile of the mechanical ventilation. We hypothesized that the temporal mechanical strain profile modulates inflammatory signalling. We applied cyclic strain with various temporal profiles to human bronchial epithelial cells (BEAS2B) and assessed proinflammatory response. The cells were subjected to sinusoidal, rectangular, or triangular strain profile and rectangular strain profile with prestrain set to 0, 25, 50, or 75% of the maximum stain, static strain, and strain resembling a mechanical ventilation-like profile with or without flow-controlled expiration. The BEAS2B response to mechanical load included altered mitochondrial activity, increased superoxide radical levels, NF-kappaB translocation, and release of interleukin-8. The response to strain was substantially modulated by the dynamics of the stimulation pattern. The rate of dynamic changes of the strain profile correlates with the degree of mechanical stress-induced cell response.

## 1. Introduction

Mechanical ventilation is an indispensable life-saving therapy that by its own nature, i.e., nonphysiological pressure conditions, is associated with the risk of tissue disruption, oxidative stress, inflammation, and pulmonary edema. As a consequence, mechanical ventilation may lead to the onset or aggravation of existing lung injury, which is then referred to as “ventilator-induced lung injury” [1]. On a cellular level, mechanical forces in terms of strain or shear stress may induce biotrauma—an extensive biological response, triggering inflammation which in turn may further aggravate the injury [1].

Despite recent improvements in lung-protective ventilation, for instance by using low tidal volumes or procedures to maintain the recruitment of the lung, morbidity and mortality associated with ventilator-induced lung injury is still unacceptably high [2].

An experimental strategy for mechanical ventilation, i.e., flow-controlled expiration (FLEX), is based on a decelerated expiration profile [3, 4]. In conventional mechanical ventilation, air is expired on behalf of the elastic properties of the respiratory system. As a result, airway pressure and lung volume drop in an exponential fashion, and in case of ARDS, the lungs empty within a few hundred milliseconds [5]. By contrast, during FLEX ventilation, the expired air is released in a decelerated fashion, and the airway pressure and lung volume decrease linearly. Clinical and experimental studies designed to estimate the effects of this ventilation mode have revealed that FLEX is able to attenuate lung injury [4], to improve homogenous lung aeration [6, 7] and arterial oxygenation [8] compared to conventional ventilation, suggesting that FLEX could provide a novel means of lung-protective ventilation.

However, with increasing evidence for the lung-protective capacity of FLEX ventilation, related to improved recruitment and higher mean airway pressure, the impact of the altered ventilation profile on a cellular level remains undetermined. We hypothesized that beyond these respiratory benefits, a decelerated stimulation profile reduces the response of the lung epithelium on cyclic mechanical strain. Therefore, we investigated the biological responses of human bronchial epithelial cells (BEAS2B) to cyclic mechanical strain, intended to mimic the mechanical strain profiles during spontaneous breathing and mechanical ventilation (to be referred to as ventilation-like strain or strain profile) with conventional and linearized expiration.

## 2. Materials and Methods

### 2.1. Cell Strain Experiments.

As a model for epithelial cell layer, we used BEAS2B (human bronchial epithelial cells, Sigma, Taufkirchen, Germany). The cells were exposed to cyclic biaxial mechanical strain using the Flexcell FX-5000 tension system (Flexcell International Corporation, Burlington, NC, USA). BEAS2B cells, cultured in RPMI 1640 medium supplemented with 5% fetal bovine serum and 1% antibiotic (all Gibco, Thermo Fisher Scientific, Waltham, MA, USA), were seeded on collagen I-coated 6-well plates (Bioflex, Flexcell International Corporation) and grown to confluence. After being washed with phosphate-buffered saline (Gibco), the cells received fresh culturing medium and were either left as controls or subjected to cyclic strain with a 20% elongation amplitude and frequency of 12 cycles per minute for 4 h (hours) with (i) sinusoidal, rectangular, or triangular cyclic strain; (ii) rectangular cyclic strain with elongation maximum of 20% and elongation minimum set to 0, 5, 10, or 15% (corresponding to 25, 50, or 75% of the maximal strain): cyclic elongation amplitudes were 20, 15, 10 or 5%, respectively; (iii) static strain of 15 or 20%; or (iv) cyclic strain following stretching profiles resembling conventional or conventional+FLEX mechanical ventilation modes [9]. Cell strain experiments were performed at 20% stretch in order to mimic ventilator-induced epithelial stretch in vitro [10]. An elongation rate of 12 cycles per minute corresponds to the normal breathing rate in humans.

Cell growth and mechanical strain experiments were carried out at standard cell-culturing conditions, i.e., 37°C, 5% CO_2_, and relative humidity >95%. At the end of the experiments, the plates were immediately placed on ice and the culture medium harvested and centrifuged at 17,000 g at 4°C for 5 min. The supernatant was then collected and stored at -80°C for later use. The cells were washed carefully with phosphate-buffered saline and fixed with 4% paraformaldehyde (Sigma).

### 2.2. Indirect Immunofluorescence Staining

For a robust fixing onto the silicone membrane and removal of cellular membranes, the cells were incubated with ice-cold methanol and acetone (both Sigma) for 10 and 1 min, respectively. NF-*κ*B was labeled with a NF-*κ*B p65 antibody (Cell Signaling Technology, Danvers, MA, USA) and visualized with an Alexa Flour 488-conjugated secondary antibody (Invitrogen, Thermo Fisher Scientific). The nuclei were counterstained with DAPI (Sigma).

### 2.3. Detection of Mitochondrial Steady State and Superoxide Radical Levels

The mitochondrial activity and superoxide radical levels in the control or stretched BEAS2B epithelial cells were detected using MitoTracker Red CM-H2XRos or dihydroethidium (DHE) dyes (Molecular Probes, Thermo Fisher Scientific) according to the manufacturer's instructions.

The fluorescence intensities were documented with an AxioImager.A1 microscope equipped with an AxioCam MRm monochromatic digital camera and Plan-Apochromat 20x/0.8 M27 lens, and evaluated using the Zen2.3 software (all Carl Zeiss Microscopy, Jena, Germany).

### 2.4. IL-8 ELISA

IL-8 quantification was performed with human IL-8 ELISA kit (Enzo Life Sciences, Inc., Farmingdale, NY, USA) according to the manufacturer's instructions. The IL-8 values were documented and evaluated with the MPM6 software and an iMark microplate reader (both Bio-Rad Laboratories, Inc., CA, USA).

### 2.5. Statistics

The experiments were performed with samples from at least three subsequent cell passages as indicated. Graphs represent means ± SD. Box plots indicate median, upper/lower quartile, and bars with minimal/maximal values. Data were analyzed using one-way ANOVA for multiple-group comparison followed by the Tukey's post hoc test, if appropriate. *P* < 0.05 was considered significant. Statistical analyses were performed with GraphPad Prism 7 (GraphPad Software, Inc., La Jolla, CA, USA).

## 3. Results

### 3.1. Effects of the Stretching Profile on Mitochondrial Steady State in BEAS2B Cells

**Figure 1:**
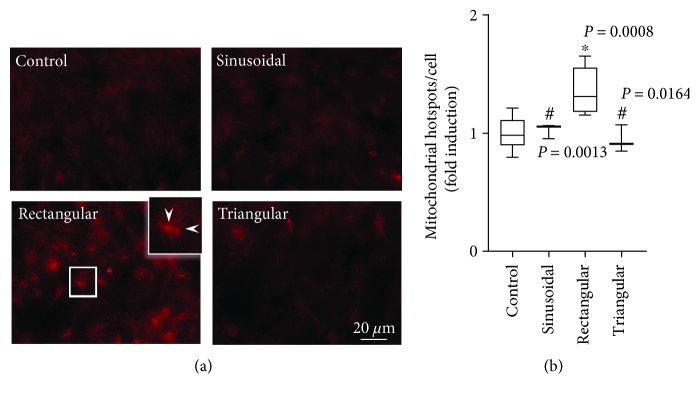
Effect of cyclic strain on mitochondrial activity. (a) Representative images of MitoTracker Red-stained control BEAS2B epithelial cells (*n* = 12) and cells subjected to sinusoidal (*n* = 3), rectangular (*n* = 6), or triangular cyclic strain (*n* = 3) for 4 h. Sites of activated mitochondria (hotspots) are exemplarily designated with white arrowheads. (b) Corresponding counts of mitochondrial hotspots per cell. Box plots indicate median, upper/lower quartile, and bars with minimal/maximal values (or median and range for *n* = 3). Statistics: analysis of variance (Tukey's post hoc test). ^∗^ *P* < 0.001 vs. control; ^#^ *P* < 0.001 vs. rectangular (unless otherwise indicated).

Staining with the MitoTracker dye revealed comparable mitochondrial activity of nonstimulated control, sinusoidally and triangularly stretched BEAS2B epithelial cells (Figure 1(a)). By contrast, rectangular strain-induced mitochondrial activity as shown by characteristic intensively stained mitochondrial patches (hotspots). Compared to controls, rectangular strain profile resulted in significantly higher hotspot counts in BEAS2B epithelial cells (Figure 1(b)).

**Figure 2:**
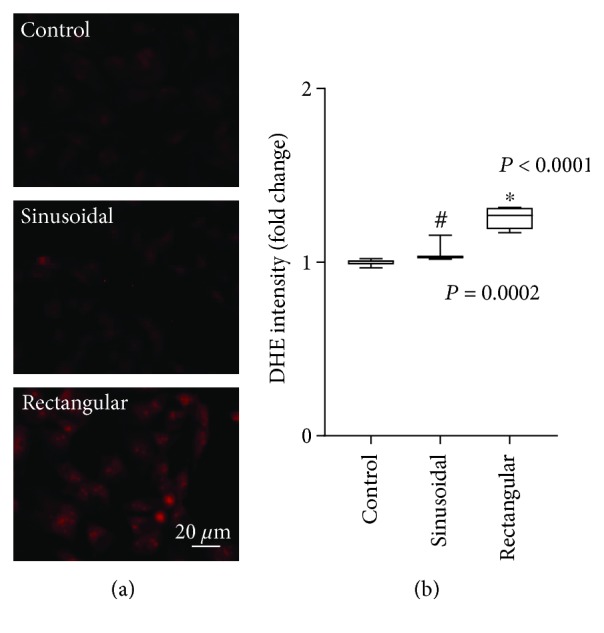
Effect of cyclic strain on superoxide radical synthesis. (a) Representative images of DHE-stained control BEAS2B epithelial cells (*n* = 6) and cells subjected to sinusoidal (*n* = 3) or rectangular (*n* = 3) cyclic strain for 4 h. (b) Corresponding densitometry analysis. Box plots indicate median, upper/lower quartile, and bars with minimal/maximal values (or median and range for *n* = 3). Statistics: analysis of variance (Tukey's post hoc test). ^∗^ *P* < 0.001 vs. control; ^#^ *P* < 0.001 vs. rectangular.

DHE staining revealed comparable superoxide radical levels of nonstimulated control and sinusoidally stretched BEAS2B epithelial cells (Figures 2(a) and 2(b)). By contrast, rectangular strain profile induced elevated oxidative stress levels as shown by characteristic and significant intensity changes.

### 3.2. NF-*κ*B Translocation

**Figure 3:**
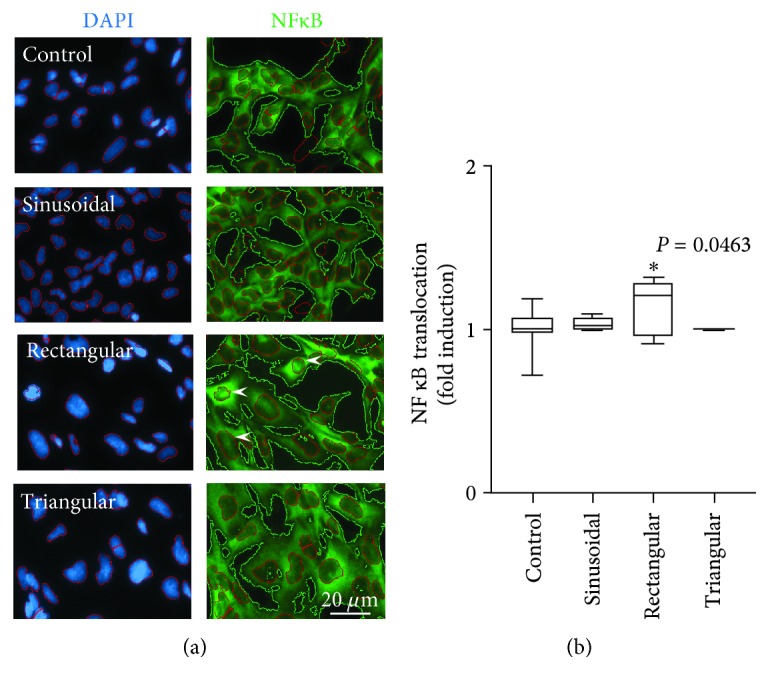
Effect of cyclic stretch on nuclear NF-*κ*B translocation. (a) Representative images of BEAS2B nonstimulated control cells (*n* = 18), cells subjected to sinusoidal (*n* = 6), rectangular (*n* = 6), or triangular (*n* = 6) cycling strain for 4 h. Left: nuclei visualized with DAPI (blue); right: NF-*κ*B distribution (green). Arrowheads indicate nuclei with elevated NF-*κ*B content. The scaling bar represents 20 *μ*m. (b) Densitometric evaluation of the ratio of total (green contours) to nuclear (red contours) NF-*κ*B signal. Box plots indicate median, upper/lower quartile, and bars with minimal/maximal values. Statistics: analysis of variance (Tukey's post hoc test). ^∗^ *P* = 0.0463 vs. control.

Nonstimulated (controls), sinusoidally and triangularly stretched BEAS2B epithelial cells showed a comparable diffuse cytosolic NF-*κ*B distribution characterized by higher perinuclear and weak nucleic density (Figure 3(a)). By contrast, rectangular strain profile resulted in elevated NF-*κ*B levels in the nuclei as suggested by the intense fluorescence signals from the corresponding areas (Figure 3(a)). Compared to controls, rectangular strain profile resulted in a significantly higher nuclear to total NF-*κ*B intensity ratio in BEAS2B epithelial cells (Figure 3(b)).

### 3.3. Effect of Mechanical Stretch Mode on IL-8 Release

**Figure 4:**
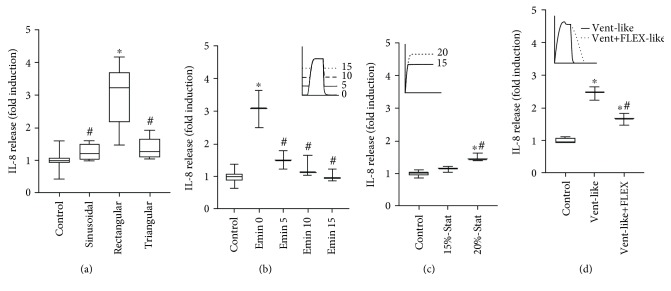
Extracellular IL-8 levels. IL-8 ELISA readings in culture medium from BEAS2B cells. (a) Nonstimulated controls (*n* = 18) and cells subjected to sinusoidal (*n* = 6), rectangular (*n* = 6), or triangular (*n* = 6) cyclic strain for 4 h. ^∗^ *P* < 0.001 vs. control; ^#^ *P* < 0.001 vs. rectangular strain profile. (b) Nonstimulated controls (*n* = 12) and cells subjected to cyclic strain (*n* = 3) with 20% elongation maximum and elongation minimum (Emin) set to 0, 5, 10, or 15%, respectively. ^∗^ *P* < 0.001 vs. control; ^#^ *P* < 0.001 vs. Emin 0. (c) Nonstimulated controls (*n* = 6) and BEAS2B cells subjected to static strain (Stat; *n* = 3) of 15 or 20%. ^∗^ *P* < 0.001 vs. control; ^#^ *P* = 0.0027 vs. 15%-Stat. (d) Nonstimulated controls (*n* = 6) or BEAS2B cells subjected to strain (*n* = 3) resembling conventional ventilation without (Vent-like) or with flow-controlled ventilation (Vent+FLEX-like). Box plots indicate median, upper/lower quartile, and bars with minimal/maximal values (or median and range for *n* = 3). Statistics: analysis of variance (Tukey's post hoc test). ^∗^ *P* < 0.001 vs. control; ^#^ *P* < 0.001 vs. Vent-like strain profile. Bars indicate mean ± SD. All strain profiles are presented in more detailed manner in supplementary figures S1-S3.

Compared to control cells, sinusoidal and triangular strain profile did not increase extracellular IL-8 release (Figure 4(a)). By contrast, rectangular strain profile resulted in significantly higher extracellular IL-8 levels compared to all other strain profiles.

BEAS2B cells subjected to 4-hour rectangular stretch with peak elongation of 20% but with group individual elongation minimum (0, 5, 10, or 15%) revealed a decreasing IL-8 release with decreasing strain amplitude (Figure 4(b)). Mere static strain applied for 4 hours also triggered IL-8 release from BEAS2B epithelial cells (Figure 4(c)).

BEAS2B epithelial cells subjected to mechanical stress with a strain profile mimicking conventional mechanical ventilation without and with flow-controlled expiration (Figure 4(d)) showed a significant increase of IL-8 levels compared to the corresponding nonstimulated control. With a flow-controlled expiration strain profile, the levels of the released IL-8 were significantly reduced compared to the conventional mechanical ventilation profile.

## 4. Discussion

Numerous experimental in vitro systems exist which, although different in their technical principle and type of the generated mechanical force, provide useful tools to study cellular response to mechanical load [11–15]. Recent research revealed the important role of intensity direction, frequency, and time of cycling strain on mechanosensitive pathways [16]. However, despite the increasing amount of data, little is known about the effects of different strain profiles in this regard. The main findings of the present study confirm our hypothesis that the biologic response to mechanical stimuli is considerably modulated by the characteristic dynamics of the strain pattern.

In our model, only rectangular mechanical strain showed distinct ROS/mitochondrial steady state pointing to differences between the stretching profiles in the initial response to mechanical load in BEAS2B epithelial cells. The relation between mechanical load and disturbed redox homeostasis is well established [17]. Increased intracellular ROS and nitric oxide as well as calcium levels were shown to occur early in the response to mechanical strain in various cell types including pulmonary epithelial cells [18–22]. It should be noted that in contrast to Chapman and colleagues [22], we did not detect elevated oxidative stress levels after 4 hours of sinusoidal cell strain. This dissimilarity stems unlikely from the different tension systems used (Flexercell 4000 vs. Flexercell 5000). While mechanical properties of epithelial cells differ [23], it is plausible to speculate that different epithelial cells might differ in their oxidative stress response to mechanical load. Further, the method used to detect oxidative stress in the present study was based on oxidation of a dihydro-X-rosamine derivate (MitoTracker Red). The MitoTracker dye oxidizes in the cytosol and importantly accumulates thereafter in the mitochondria depending on the mitochondrial membrane potential. The MitoTracker staining may provide an estimate of mitochondrial homeostasis [24]. Further, specific to superoxide radicals, dihydroethidium stain [25] substantiated that altered mitochondrial activity results in elevated oxidative stress. Moreover, both the mitochondrial steady state and ROS are sensitive to mechanical load [26, 27].

ROS and mitochondrial steady state might contribute to NF-*κ*B activation during mechanical stress [28, 29]. Latent NF-*κ*B is found in the cytosol but transported into the nuclei upon activation [30]. Active NF-*κ*B plays a critical role in various cellular processes associated with proliferation, cell death, development, and innate and adaptive immune responses [31]. In contrast to sinusoidal or triangular cyclic strain, rectangular strain profile could apparently trigger NF-*κ*B activation in BAES2B cells. Mechanical strain-induced NF-*κ*B activation is related to overexpression of various proinflammatory cytokines, e.g., IL-1*β*, IL-6, IL-8, and TNF*α* [32]. Proinflammatory signaling was proven in different lung relevant cells [9, 33, 34].

Particularly, IL-8 overexpression and release appeared as reliable indicators of cell stress related to static or cyclic mechanical strain in A549 and BEAS2B epithelial cells [9, 33]. Taking cytokine signaling is crucial in terms of onset and development of inflammation, we further analyzed the IL-8 response as an “essential” and “decisive” indicator to estimate the impact of the temporal strain profile on BEAS2B epithelial cells. Consistent with the previous findings [33], rectangular cell strain induced IL-8 release in BEAS2B cells. Time course experiments (supplementary Fig.  S4) revealed that after 4 hours of mechanical strain, extracellular IL-8 levels were at least three times higher than in the time-matched unstrained controls. Comparison between strain modes revealed that altered ROS/mitochondrial steady state and assumed activation of NF-*κ*B correlated with the extracellular IL-8 levels as observed after 4 hours of mechanical strain. In this respect, only rectangular mechanical strain appeared strong enough to induce proinflammatory signaling in BEAS2B cells.

A pure mechanistic explanation of the observed relation between the strain profile and the rate of stress response could be provided from a signal analysis point of view: each strain profile may be seen as composed of a combination of sinusoidal time curves. Thereby, a smoother signal contains lesser high-frequency components. For example, a sinusoidal profile consists of a single sine wave whereas an ideal rectangular profile consists of a sine wave of the main frequency plus infinite harmonics. Of course, real signals differ from ideal ones. However, the Fourier analysis of the rectangular temporal profile showed a number of high-frequency components that were either missing or appeared with different amplitude in sinusoidal and triangular and between “ventilation-like” and “ventilation-like+FLEX” profiles (supplementary Fig.  S1 and S2, respectively). These high-frequency components contain additional energy, and consequently, rapid transitions provide more physical load transferred to the sample [35]. With respect to the translational meaning of our results, it is important to consider that ventilator-induced lung injury may develop if the mechanical power input exceeds a certain threshold [36]. Indeed, the sinusoidal and triangular profiles are clearly smoother in their transitions between minimum and maximum elongation, compared to the rectangular profile. This suggests that not only the stimulation amplitude but also the rapidity of the transition of strain, distinct for the rectangular profile, contributes to the onset of the stress response. The proposed possibility finds additional proof in the direct comparison between strain profiles mimicking mechanical strain during volume-controlled ventilation with or without controlled expiration. The two profiles differed only in the rate of relaxation, and the decelerated profile induced less proinflammatory response.

Further, the stress response on rectangular strain profile diminished with increasing strain minimum (25, 50, or 75% of maximal strain), which was associated with a decreasing elongation amplitude. This set of experiments was designed to mirror mechanical strain during positive end-expiratory pressure (PEEP) ventilation. Although the lung protective potential of PEEP is still under evaluation [37], our in vitro results reflect the concept of the “driving pressure” [38] (only reflecting a PEEP increase combined with maintenance of the peak pressure) supporting the possibility that mechanical ventilation at higher PEEP might be less important for strain-induced proinflammatory signaling than the “absolute” amplitude of the cyclic mechanical load. Presumably, BEAS2B epithelial cells might adapt to mechanical strain with lower amplitudes. Alternatively, cells might use different mechanisms to sense static and dynamic strain [39], which subsequently might lead to different or delayed inflammatory response. The later suggestions implicated that the proinflammatory response may not be solely related to the amount of energy released from a particular strain profile but may as well depend on the biological properties of the cells.

Taken together, the presented data reveal that mechanical load-triggered inflammatory signaling is apparently modulated by the added effects of all profile components (e.g., extension, amplitude, hold, and relaxation) and the specific dynamics of a particular strain profile. The deceleration/linearization of strain is sufficient to reduce the inflammatory stress response. In this respect, one may speculate if the above-mentioned lung-protective effects of flow-controlled expiration could be supported by a diminished inflammation in the epithelial cells.

The presented results suggest that the dynamic changes of the strain profiles may predetermine mechanical stress-induced proinflammatory response. However, the potential of decelerated mechanical ventilation to reduce the risk for strain-related inflammation has to be addressed in future studies.

## 5. Conclusion

In vitro models for investigating the effects of cyclic mechanical strain are useful tools to study the impact of mechanical ventilation settings on lung-specific cells. Our findings demonstrate profile-dependent differences in the cellular response to mechanical strain. Oxidative stress, NF-*κ*B translocation, and IL-8 release in BEAS2B epithelial cells are intimately related to the dynamics of mechanical strain.

## Data Availability

All data used to support the findings of this study are included within the article (main body and supplement). Raw data can be available on request after publication.

## Supplementary Materials

Supplementary Materials Fig. S1: frequency analysis of sinusoidal rectangular and triangular strain profiles. A: temporal courses of the applied elongation profiles. B: the frequency components of the applied strain profiles, normalized on the main frequency. The rectangular strain profile showed the largest portions of high-frequency components; the sinusoidal strain profile showed the smallest portions of high-frequency components. Fig. S2: frequency analysis of resembling conventional ventilation without (Vent-like) or with flow-controlled ventilation (Vent-like+FLEX) strain profiles. A: temporal courses of the applied elongation profiles. B: the frequency components of the applied strain profiles, normalized on the main frequency. The rectangular strain profile showed the largest portions of high-frequency components; the sinusoidal strain profile showed the smallest portions of high-frequency components. Fig. S3: A: strain profiles of 20% elongation maximum and elongation minimum set to 0, 5, 10, or 15%. B: profiles of static strain set to 15 or 20% elongation. Fig. S4: time courses of IL-8 release from BEAS2B epithelial cells subjected to rectangular cyclic strain for 3, 4, 6, and 8 h and nonstimulated control. Bars indicate mean ± SD (*n* = 3/group). ELISA reads of strained cells were presented as fold induction for each time-matching control. *T*-test. ^∗^ *P* < 0.05 vs. time-matching control.
