# Supplementary material for: Ventilation-Like Mechanical Strain Modulates the Inflammatory Response of BEAS2B Epithelial Cells
Source: Oxid Med Cell Longev. 2019 Jun 19;2019:2769761. doi: 10.1155/2019/2769761 (PMC6607724; doi:10.1155/2019/2769761)
Supplement: Supplementary Materials — Fig. S1: frequency analysis of sinusoidal rectangular and triangular strain profiles. A: temporal courses of the applied elongation profiles. B: the frequency components of the applied strain profiles, normalized on the main frequency. The rectangular strain profile showed the largest portions of high-frequency components; the sinusoidal strain profile showed the smallest portions of high-frequency components. Fig. S2: frequency analysis of resembling conventional ventilation without (Vent-like) or with flow-controlled ventilation (Vent-like+FLEX) strain profiles. A: temporal courses of the applied elongation profiles. B: the frequency components of the applied strain profiles, normalized on the main frequency. The rectangular strain profile showed the largest portions of high-frequency components; the sinusoidal strain profile showed the smallest portions of high-frequency components. Fig. S3: A: strain profiles of 20% elongation maximum and elongation minimum set to 0, 5, 10, or 15%. B: profiles of static strain set to 15 or 20% elongation. Fig. S4: time courses of IL-8 release from BEAS2B epithelial cells subjected to rectangular cyclic strain for 3, 4, 6, and 8 h and nonstimulated control. Bars indicate mean ± SD (n = 3/group). ELISA reads of strained cells were presented as fold induction for each time-matching control. T-test. ∗ P < 0.05 vs. time-matching control. [file 2769761.f1.docx]

Supplementary Materials:


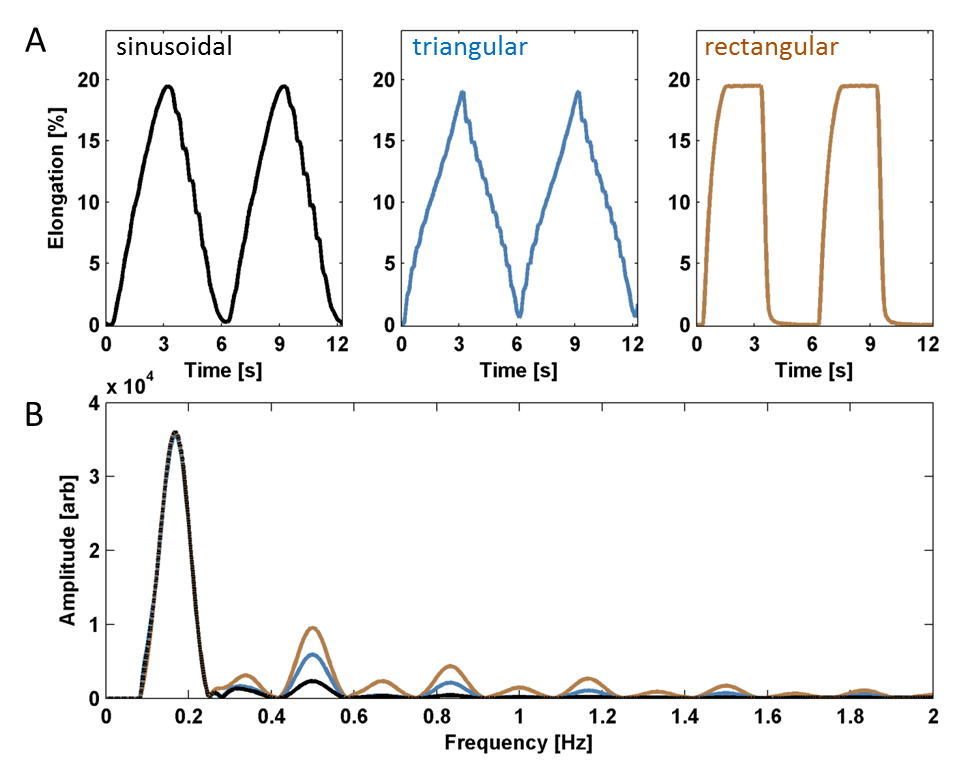


**Supplementary Fig. S1** Frequency analysis of sinusoidal rectangular and triangular strain-profiles. A: Temporal courses of the applied elongation profiles. B: The frequency components of the applied strain profiles, normalized on the main frequency. The rectangular strain-profile showed the largest portions of high frequency components, the sinusoidal strain-profile showed the smallest portions of high frequency components.

**
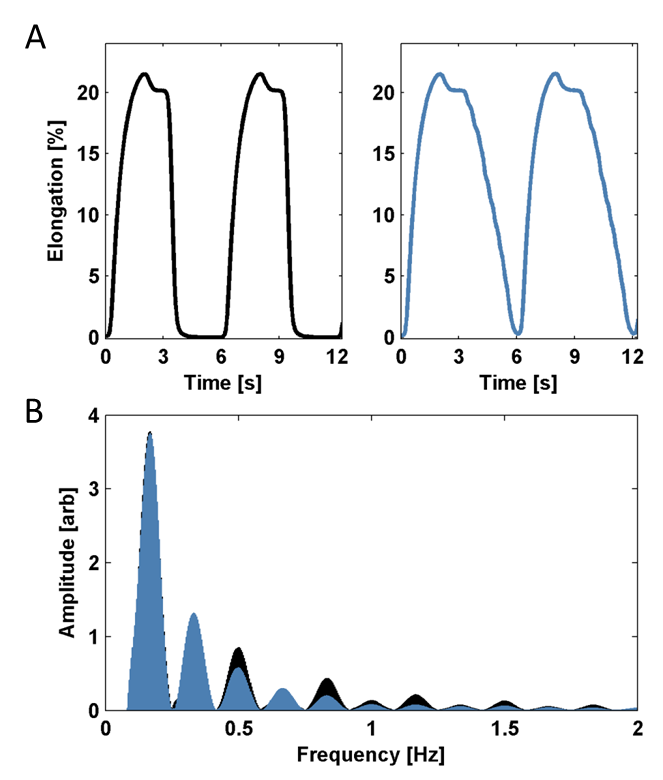
**

**Supplementary Fig. S2** Frequency analysis of resembling conventional ventilation without (Vent-like) or with flow controlled ventilation (Vent-like+FLEX) strain-profiles. A: Temporal courses of the applied elongation profiles. B: The frequency components of the applied strain-profiles, normalized on the main frequency. The rectangular strain-profile showed the largest portions of high frequency components, the sinusoidal strain-profile showed the smallest portions of high frequency components.


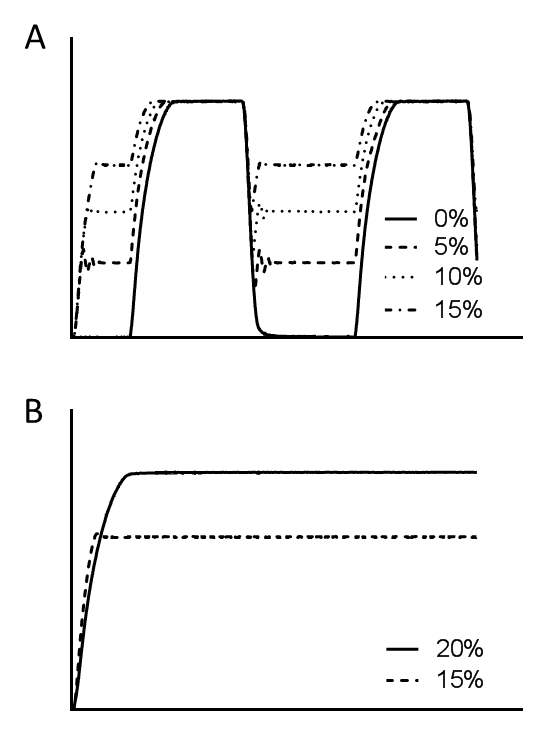


**Supplementary Fig. S3** A Strain profiles of 20% elongation maximum and elongation minimum set to 0, 5, 10 or 15%. B Profiles of static strain set to 15 or 20% elongation.


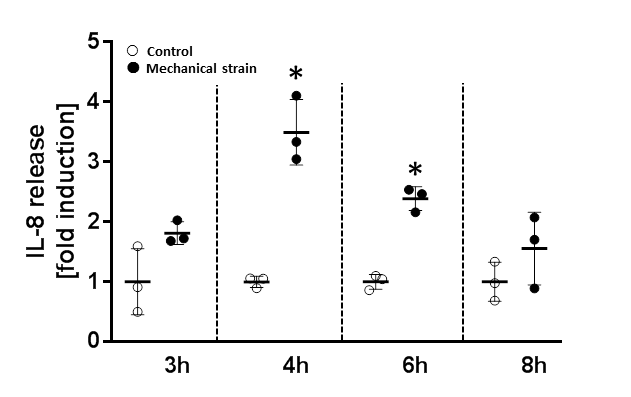


**Supplementary Fig. S4** Time courses of IL-8 release from BEAS2B epithelial cells subjected to rectangular cyclic strain for 3,4,6,8 h and non-stimulated control. Bars indicate mean +/- SD (n=3/group). ELISA reads of strained cells were presented as fold induction for each time matching control. T-test. * p< 0.05 vs time matching control.
